# Identification of the first C1 subgenotype of enterovirus 71 in the Chinese mainland in a retrospective study

**DOI:** 10.1186/s12985-022-01810-5

**Published:** 2022-05-15

**Authors:** Fenfen Si, Dongyan Wang, Tianjiao Ji, Yong Zhang, Shuangli Zhu, Junhan Li, Wenbo Xu, Zexin Tao, Dongmei Yan

**Affiliations:** 1 National Polio Laboratory, WHO WPRO Regional Polio Reference Laboratory, National Health Commission Key Laboratory for Biosecurity, National Health Commission Key Laboratory for Medical Virology, National Institute for Viral Disease Control and Prevention, Chinese Center for Disease Control and Prevention, Beijing, 102206 China; 2 Beijing Fengtai District Center for Disease Control and Prevention, Beijing, 10071 China; 3 Center for Biosafety Mega-Science, Chinese Academy of Sciences, Wuhan, 430000 China; 4 Shandong Center for Disease Control and Prevention, Jinan, 250014 China

**Keywords:** C1 sub-genotype of Enterovirus 71, Retrospective study, Guillain–Barre syndrome, Acute Flaccid Paralysis

## Abstract

The C4 sub-genotype of Enterovirus 71 (EV71) has been identified as the most dominant sub-genotype circulating in the Chinese mainland since 1998. The circulation situation of EV71 before 1998 is not well established due to insufficient experimental data. The C1 subgenotype of EV71 has not yet been reported in the Chinese mainland by now. Based on the AFP surveillance system of the mainland of China, this study conducted a retrospective study of AFP cases for 1985–1999: a strain of EV-A71 C1 subgenotype was found. To our knowledge, this strain (SD92-41) is the first C1 sub-genotype reported in the Chinese mainland. This study demonstrates that the C1 gene subtype also appeared in the Chinese mainland, but it is unknown whether it is an imported or a local epidemic strain. With sufficient information known from retrospective studies, the source of the SD92-41 strain will be identified and the prevalence of EV-A71 in the Chinese mainland before 1998 will be clearer.

## Introduction

As a member of the Human Enterovirus species A (genus *Enterovirus*, family *Picornaviridae*), Enterovirus 71 (EV71) is a small, non-enveloped, positive-stranded RNA virus. Based on the VP1 coding region, the worldwide circulation of EV71 can be divided into seven genotypes designated A to G, where the two major genotypes B and C are classified into 14 subgenotypes, respectively, designated B0 to B7 and C1 to C6 [1]. The sub-genotype C4 has been identified as the most dominant sub-genotype circulating in the Chinese mainland since 1998. The circulation situation of EV71 before 1998 is not well established due to insufficient experimental data [2]. It is reported that the sub-genotype C1 of EV71 circulated only in the Western Pacific, Europe, and the United States before 2000. After 2000, it was reported that the C1 genotype was prevalent in Southeast Asia, such as Malaysia, Thailand, Hong Kong. However, the sub-genotype C1 of EV71 has not been reported in the Chinese mainland by now [3]. China established the AFP (Acute Flaccid Paralysis, AFP) surveillance system to monitor acute onset cases, decreased muscle tension, decreased muscle strength, and weakened or disappeared tendon reflex. Based on the AFP surveillance system of the mainland of China, this study performed a retrospective study of AFP cases for 1985–1999: A strain of EV-A71 C1 sub-genotype was found.

## Material and methods

172 human rhabdomyosarcoma (RD) cell-positive virus isolates from the AFP surveillance system in the Chinese mainland from 1985 to 1999 were retrospectively studied, and a molecular typing method was performed. Primer pair Y7/Q8 was used for poliovirus screening. Primer pairs 486/488 and 040/011 were used for the isolates with negative results to amplify the partial VP1 sequences. The combination of the two sequences yielded the entire VP1 coding region. 164 polioviruses and 8 (4.7%) NPEV strains were identified. 8 NPEVs included 2 Coxsackievirus B5, 2 Echovirus 1, 1 EV71, 1 Echovirus 26, 1 Coxsackievirus B1, and 1 Enterovirus-B.

The VP1, P1, P2, P3 coding region of the EV71 strain (SD92-41) were aligned with EV-A prototype strains and EV71 typical strains using the MUSCLE algorithm implemented via MEGA v7.0, respectively. Maximum likelihood trees were constructed using the GTR + G model and were implemented in MEGA7.0 with 1000 bootstrap replicates.

## Results

**Fig. 1:**
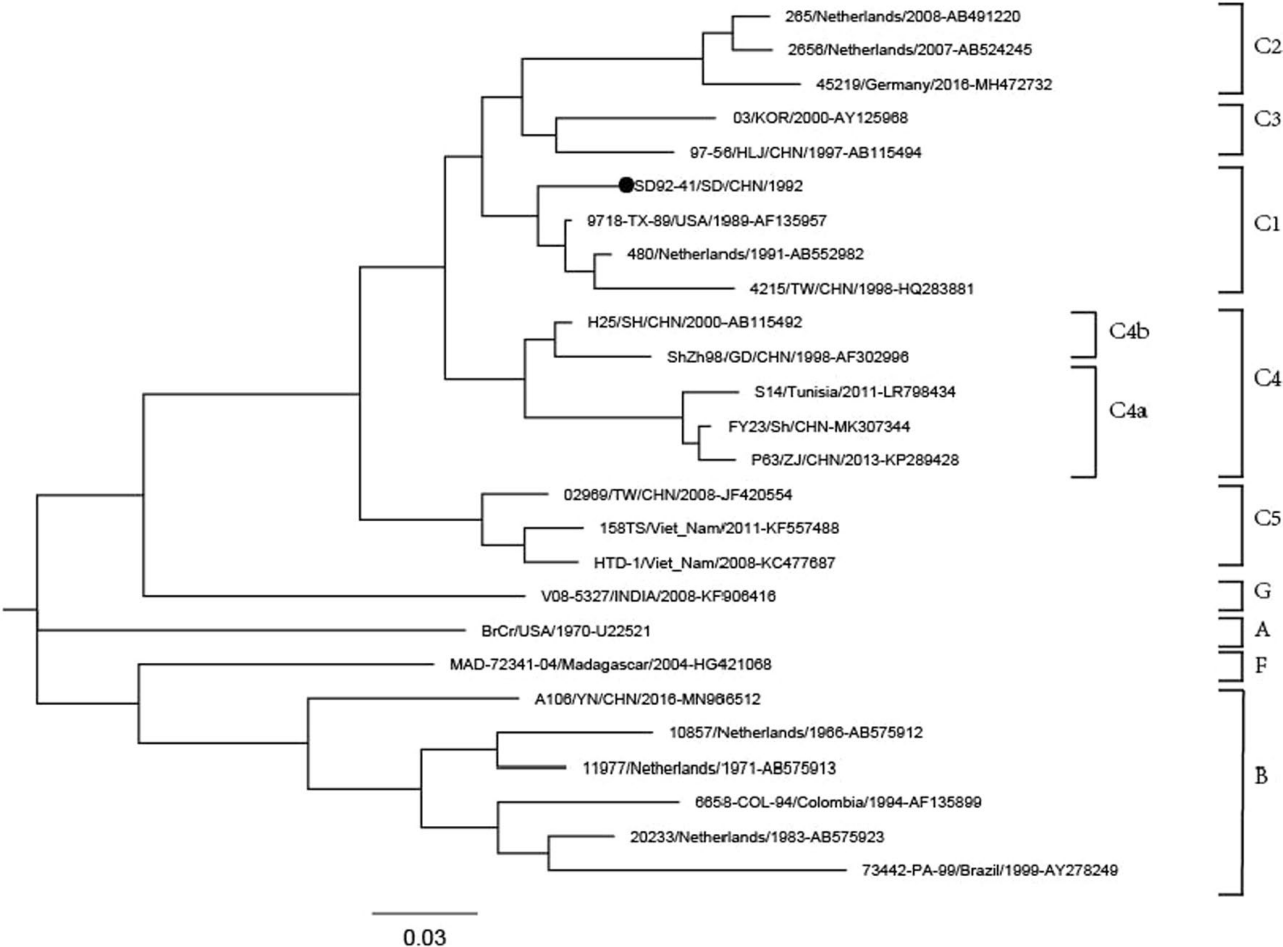
Phylogenetic Tree Based on Entire VP1 Coding Regions of EV71. *Note* The SD92-41 strain is marked with a black solid circular

The EV71 strain (SD92-41) was isolated from a 7-year-old male patient with AFP in Shandong Province in 1992. The AFP case was diagnosed as Guillain–Barre syndrome (GBS) by a polio diagnosis expert panel, and the patient had no residual paralysis during the 60-day clinical follow-up. Phylogenetic analysis based on entire VP1 coding regions of EV71 was conducted with the maximum-likelihood (ML) method revealed that the SD92-41 strain belonged to the C1 sub-genotype (Fig. 1). The VP1 genome sequence of the SD92-41 strain and the EV-71 prototype strain showed 96.9% similarity in nucleotide sequence and 80.8% similarity in amino acid sequence. The homologous comparison revealed that the VP1 genome sequence of the SD92-41 strain had the highest homology with the strain 9718-TX-89 from the U.S. in 1989 and had 99.7% similarity in nucleotide 96.2% similarity in amino acid sequence.

**Fig. 2:**
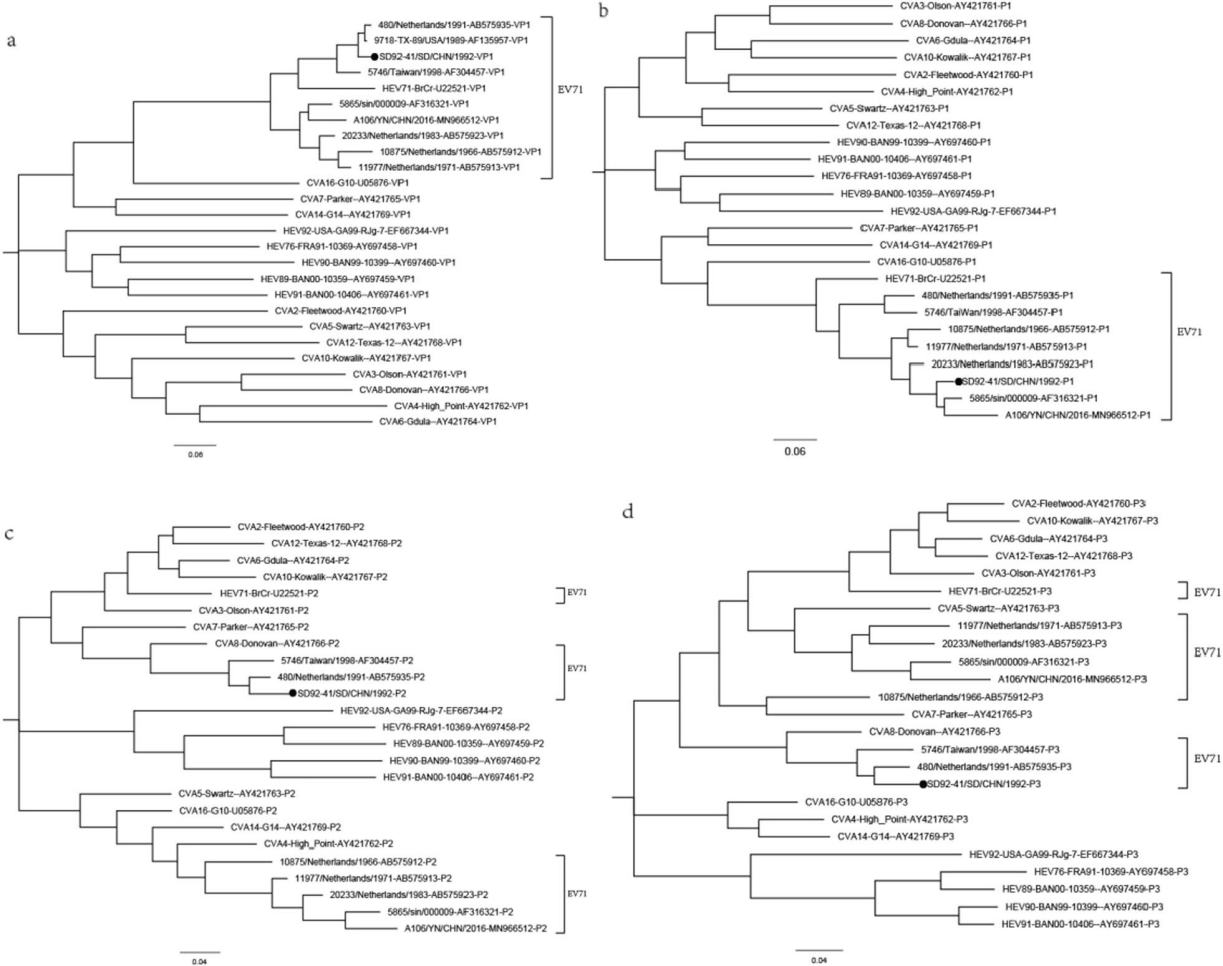
Phylogenetic Tree Based on EV-A's VP1, P1, P2, and P3 sequences. *Note* Maximum likelihood trees were constructed using the GTR + G model and were implemented in MEGA7.0 with 1000 bootstrap replicates. The close circle represents the SD92-41 strain. The scale bars indicate the genetic distance. **a** VP1 coding sequence; **b** P1 coding sequence; **c** P2 coding sequence; **d** P3 coding sequence

**Fig. 3:**
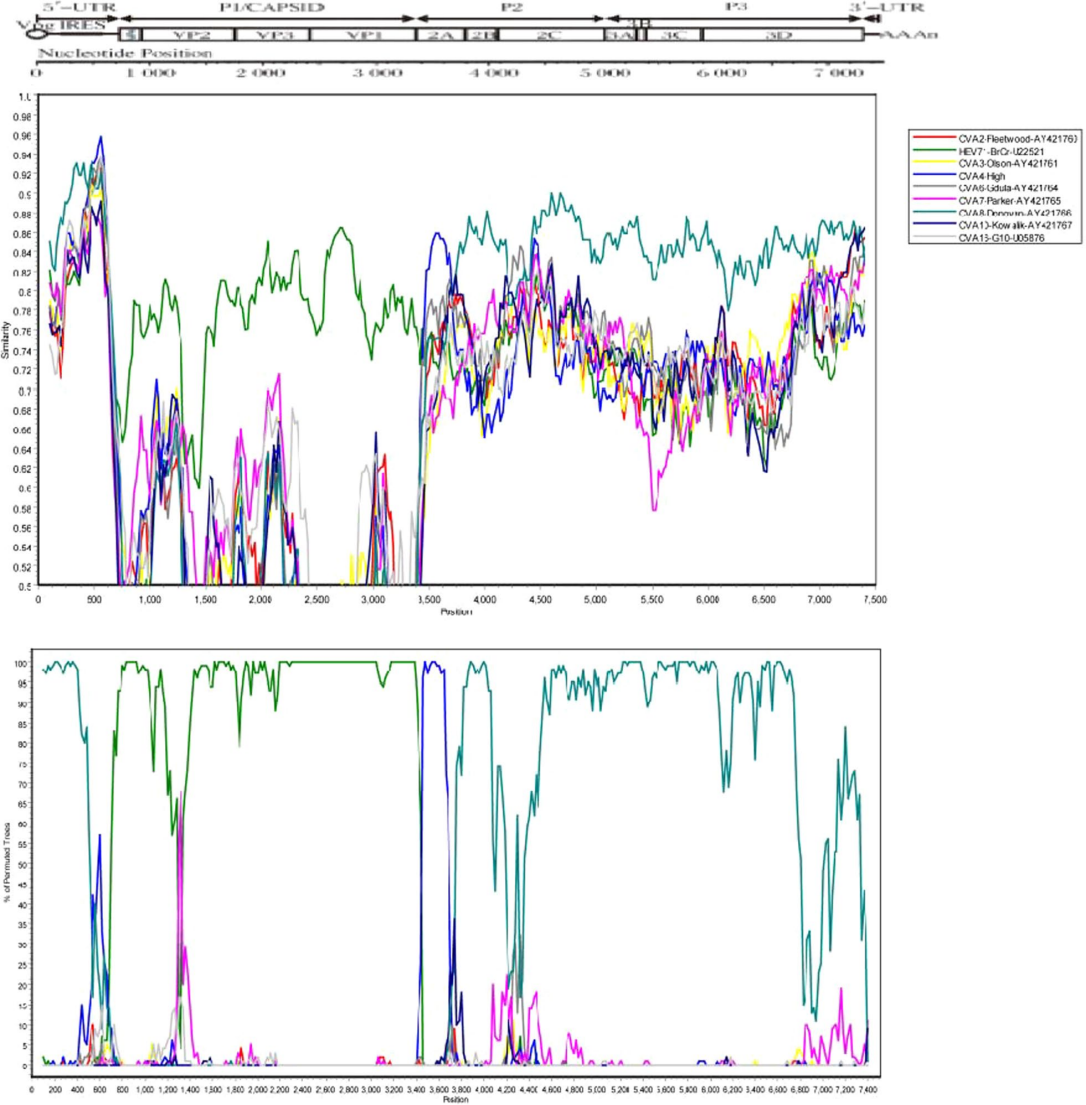
Similarity and bootscanning analysis of the SD92-41 strain and other EV-A strains. The SD92-41 strain was used as a query sequence

The whole-genome sequence of the SD92-41 strain was determined to be 7411 nt long. The ORF of the SD92-41 strain is 6582 nt in length, encoding a polypeptide of 2193 amino acids, with a 746nt 5’-UTR and an 83nt 3’-UTR. Phylogenetic trees based on VP1, P1, P2, P3 coding regions of prototypes strains of the human enterovirus species A and EV71 typical strains were constructed with the maximum-likelihood (ML) method (Fig. 2). The topology of the phylogenetic tree based on the nucleotide sequences of the P1 region was similar to that of the VP1 region. The SD92-41 strain and other sub-genotypes of EV71 were clustered into a large cluster (Fig. 2a, b). However, SD92-41 and CVA8 were clustered into one branch in P2 and P3 regions, indicating that SD92-41 and CVA8 may be recombined. Simplot analysis showed that SD92-41 and CVA8 had gene recombination highly in P2 and P3 regions (Fig. 3). It is worth noting that SD92-41 and the other two EV71 strains (480/Netherlands/1991-AB115492 and 5746/Taiwan/1998-AF304457) were clustered in the same branch in P2 and P3 regions, which means that two EV71 strains have high similarity to SD92-41 in the P2 and P3 regions.

## Discussion

Since 1998, the C4 subgenotype strains of Enterovirus 71 have been persistently circulating in the Chinese mainland for 22 Years [2], where only one or two imported subgenotypes were reported [4]. Reports on the circulation situation of EV71 before 1998 are few. One sequence from Heilongjiang in 1997 and one from ShanDong in 1996 were identified as C3 and C2 subgenotypes, respectively, indicating that the C3 and C2 subgenotypes appeared in mainland China [5]. To our knowledge, SD92-41 is the first C1 subgenotype reported in the Chinese mainland, which showed high sequence similarity in the VP1 region to the strain 9718-TX-89 from the U.S. in 1989, but there is no direct evidence supporting the association with the American strain. SD92-41 has a sequence homology in the whole genome region to the two other EV71 strains (480/Netherlands/1991-AB115492 and 5746/Taiwan/1998-AF304457) inferred from the experimental fact that the recombination events might occur first in the common ancestor and then they spread to different countries. This study proves that the C1 gene subtype also appeared in the Chinese mainland, but the source of the SD92-41 strain is not clear because of insufficient information.

## Data Availability

Condensed anonymized data are available from the corresponding author on reasonable request. Whole-genome nucleotide sequences for the strain determined in this study have been deposited in the GenBank nucleotide sequence database under accession numbers MW473684.

## Abbreviations

EV71: Enterovirus 71
AFP: Acute Flaccid Paralysis
RD: Rhabdomyosarcoma
GBS: Guillain–Barre syndrome
